# Heat Shock Protein 90 in Alzheimer's Disease

**DOI:** 10.1155/2014/796869

**Published:** 2014-10-13

**Authors:** Jiang-Rong Ou, Meng-Shan Tan, An-Mu Xie, Jin-Tai Yu, Lan Tan

**Affiliations:** ^1^Department of Neurology, Qingdao Municipal Hospital, School of Medicine, Qingdao University, No. 5 Donghai Middle Road, Qingdao 266071, China; ^2^Department of Neurology, Qingdao Municipal Hospital, College of Medicine and Pharmaceutics, Ocean University of China, Qingdao 266003, China; ^3^Department of Neurology, the Affiliated Hospital of Qingdao University, Qingdao 266000, China

## Abstract

Alzheimer's disease (AD) is the first most common neurodegenerative disease. Despite a large amount of research, the pathogenetic mechanism of AD has not yet been clarified. The two hallmarks of the pathology of AD are the extracellular senile plaques (SPs) of aggregated amyloid-beta (A*β*) peptide and the accumulation of the intracellular microtubule-associated protein tau into fibrillar aggregates. Heat shock proteins (HSPs) play a key role in preventing protein misfolding and aggregation, and Hsp90 can be viewed as a ubiquitous molecular chaperone potentially involved in AD pathogenesis. A role of Hsp90 regulates the activity of the transcription factor heat shock factor-1 (HSF-1), the master regulator of the heat shock response. In AD, Hsp90 inhibitors may redirect neuronal aggregate formation, and protect against protein toxicity by activation of HSF-1 and the subsequent induction of heat shock proteins, such as Hsp70. Therefore, we review here to further discuss the recent advances and challenges in targeting Hsp90 for AD therapy.

## 1. Introduction

Alzheimer's disease (AD) is the most common progressive neurodegenerative disorder that is characterized by the formation of extracellular accumulation of amyloid-*β* (A*β*) in senile plaques and intracellular neurofibrillary tangles (NFTs) [1]. A*β* is a protein fragment generated from an amyloid precursor protein (APP); the fragments accumulate to form hard, insoluble plaques. The NFTs are composed of microtubule-associated protein tau. The microtubules are the main structures in the maintenance of neuronal morphology. The tau protein is abnormal and the microtubule structures collapse in AD [2].

Molecular chaperones assist the folding of newly synthesized or misfolded proteins, preventing their aggregation [3]. Heat shock protein 90 (Hsp90), as a molecular chaperone, is capable of suppressing protein aggregation, solubilizing protein aggregates, and targeting protein clients for degradation. HSP-induced microglial activation may serve in neuroprotective role by facilitating A*β* clearance and cytokine production in AD. Kakimura et al. found that extracellular heat shock proteins (HSPs), such as Hsp90, Hsp70, and Hsp32, may facilitate A*β* clearance by the activation of microglial phagocytosis and A*β* degradation by NF-*κ*B and p38 MAPK activation through the Toll-like receptor-4 (TLR4) pathway [4]. In addition, the high-affinity Hsp90-CHIP complex recognizes and selectively degrades phosphorylated tau client proteins in AD, a critical mediator of this mechanism is carboxy terminus of Hsp70-interacting protein (CHIP), a tau ubiquitin ligase. Cochaperones were also involved in Hsp90-mediated removal of p-tau, while those of the mature Hsp90 refolding complex prevented this effect. It may provide a rationale for the development of novel Hsp90-based therapeutic strategies [5]. So, Hsp90 may be an attractive choice for therapeutic targeting in AD and would be recognized as a strong potential therapeutic target in suppressing or curing AD [2, 6–8]. In this paper, we review the recent knowledge on Hsp90 in AD and will focus on the potential advances and challenges in targeting Hsp90 for AD therapy.

## 2. Hsp90: Structure and Function

HSPs are highly conserved proteins found in fungi, animals, and plants [4, 9, 10]. HSPs synthesis can be induced by stressful conditions, such as heat shock, ischemia, hypoxia, heavy metals, and amino acid analogs. Mammalian HSPs have been divided into several families in accordance with molecular weight, including Hsp110, Hsp90, Hsp70/Hsp80, Hsp60, and small molecular HSP (smHSP) [2]. Hsp90, a highly conserved molecular chaperone present from bacteria to mammals, is one of the most abundant proteins in prokaryotic and eukaryotic cells, accounting for 1~2% of total proteins. They are essential for viability in eukaryotes [10, 11]. In mammalian cells there are two Hsp90 subtypes: Hsp90α and Hsp90*β* [12]. Hsp90α and Hsp90*β* are 85% identical and both are in the cytosol [3]. Hsp90α is sensitive to stress conditions, whereas Hsp90*β* is constitutively expressed [13]. The major functions of Hsp90α are protein transportation and folding, as well as maintenance of normal conformation, while Hsp90*β* is related to the utilization of steroid hormones [10].

Structurally, Hsp90 is a conformationally dynamic dimeric protein consisting of three conserved domains, an N-terminal ATP-binding domain (N-domain), a middle domain (M-domain), and a C-terminal dimerization domain (C-domain) [13, 14]. The N-terminal domain (Hsp90-N) contains the ATP/ADP binding site with intrinsic ATPase activity [15]. It is involved in conformational changes and is conducive to signal transduction, protein folding, and morphological evolution. Several cochaperones, including p23, Cdc37, Aha1, and Sgt-1 [16, 17], and substrate binding properties have also been related to Hsp90-N [12, 18, 19]. Conformational transitions of Hsp90 between an open conformation with the N-domains separated and a closed conformation with the N-domains associated depend on ATP binding and hydrolysis [10, 20, 21]. The middle domain (Hsp90-M) plays an important role in activating the ATP hydrolysis in Hsp90-N and contributes to the interaction sites for client proteins and some cochaperones [12, 14, 16]. The function of Hsp90 depends not only on the N-terminal domain, but also on the participation of the middle domain [10, 22]. The C-terminal domain (Hsp90-C) is essential for a dimerization domain of Hsp90. It was suggested to contribute to the site of calmodulin and substrates binding [12, 23, 24]. In eukaryotic cytoplasm, the Hsp90-C contains a conserved five peptide domain (MEEVD) that is recognized by a tetratricopeptide repeat (TPR) domains of several cochaperones, some of which link the Hsp90 system to the HSP70 system and the proteasome [12, 25, 26].

It has been found in two extreme conformational states in the Hsp90 dimer, a “tense” state and a “relaxed” state. The former causing the formation of a closed structure is combined with ATP to the N-terminal domains, and the latter in which the N-terminal domains are dissociated and the dimer has a V-shaped structure [27, 28].

At present, the biological functions of Hsp90s are supposed to mainly involve assisted protein folding, complex assembly, and degradation, as well as activation of substrates or enhancing the biological activity of substrate proteins [10, 29, 30]. Hsp90 mainly interacts with three types of proteins in signal transduction, including cochaperones, regulatory factors, and substrate proteins. Hsp90 proteins require cochaperones and regulatory factors to regulate the relation between themselves and substrate proteins, which together confer the diverse physiological activities of Hsp90 [10]. Cochaperones play a major role in regulating the ATP enzymatic activity of Hsp90s in cytoplasm and in mediating interactions between Hsp90s and substrates [10, 31, 32]. To date, more than 20 cochaperones have been identified. They regulate the function of Hsp90 by inhibiting and activating the ATPase of Hsp90 and recruiting the specific client proteins to the cycle in different ways [13, 33, 34]. Hsp90s also affect the folding and activation of a wide variety of substrate proteins, most of which are kinases and transcription factors involved in signal transduction and regulatory processes [10, 35].

Furthermore, Hsp90 functions include some complexes of chaperone proteins such as HSP70, Hop, Cdc37, and p23 [36]. One of Hsp90 complexes directs Hsp90 client proteins to proteasome degradation by proteasome targeting with HSP70 and Hop, while the other complex stabilizes Hsp90 client proteins by form with Cdc37 and p23 [37, 38]. Hsp90 client proteins may also be recognized initially by the HSP40/HSP70 complex with CHIP and transferred to the Hsp90 complex by Hop [37].

## 3. Possible Roles of Hsp90 in AD

### 3.1. Hsp90 and A*β*

One of the pathological characteristics of AD is the accumulation of fibrillar A*β* peptides to form amyloid plaques in the nerve cells in the brain. A*β* is a protein fragment generated from an amyloid precursor protein (APP). APP is a membrane-associated protein and is processed through the endoplasmic reticulum (ER) and Golgi complex [39]. A*β* accumulates to form hard, insoluble plaques in AD. Both soluble and insoluble A*β* species are considered to be responsible for initiating the pathological cascade that eventually leads to AD [40]. This A*β* peptide has amyloidogenic properties, forming multimers in a *β*-sheet structure. As enough of this material is produced, amyloid plaques form spontaneously and remain quite stable over time [39, 41]. Microglia are macrophage-like phagocytic cells in the brain. In AD, extracellular A*β* (1-40, 1-42, or 1-43) deposits are markedly accumulated and associated with microglia responses [4]. Microglial phagocytosis is more sensitive to A*β*(1-42) peptides and that the clearance of phagocytosed A*β*(1-42) peptides may be mediated by microglial peptidases such as insulin-degrading enzyme (IDE, a thiol metalloendopeptidase) and cathepsin D. HSPs increase the competence of microglia to phagocytose monomer and aggregated A*β*(1-42) peptides and then degrade these peptides [42].

HSPs induced the production of interleukin 6 (IL-6) and tumor necrosis factor α (TNFα) and increased the phagocytosis and clearance of A*β* peptides. The mechanism of microglial activation by exogenous HSPs involves the NF-*κ*B and p38 mitogen-activated protein kinase pathways mediated by Toll-like receptor 4 activation. Hsp90-induced production of IL-6 or TNFα was inhibited by inhibitors of both NF-*κ*B and p38 MAPK, which showed that HSP-induced activation of both NF-*κ*B and p38 MAPK pathways may contribute to cytokine production [4].

The complex of Hsp90 and HSP70/HSP40 also can inhibit A*β* formation and slow the rate of aggregation in a chaperone concentration-dependent manner [43]. In addition, it was necessary for inhibition of protein aggregation for the HSP40/HSP70 and Hsp90's ATPase activity. There are two mechanisms by which HSPs inhibit A*β* assembly have been proposed. In one pattern, the chaperone binds misfolded amyloid in an ATP-independent manner, preventing it from aggregation. This pattern is consistent with the observed dependency on Hsp90 ATPase activity. Alternatively, the chaperone may bind A*β* in an ATP-dependent manner, changing A*β* conformation to one that is less susceptible to aggregation [43, 44].

Additionally, cytosolic HSP70 and Hsp90 were shown to inhibit early stages of amyloid aggregation. Evans et al. had thought that there were two specific models to inhibit A*β* self-assembly. In the “holding” model, the chaperone binds misfolded amyloid (likely via its substrate-binding domain). In the “refolding” model, chaperones not only bind A*β* but also change its structure. The altered structure is defined as being less competent for progression through the aggregation pathway [43].

### 3.2. Hsp90 and Tau

The other hallmark of the pathology of AD is the accumulation of the microtubule-associated protein tau, which leads to the formation of toxic NFTs [5, 45]. NFTs are insoluble and twisted fibers found inside the brain's cells [2, 46]. Tau phosphorylation and aggregation have been related with conformational changes of the protein. In AD, hyperphosphorylation of the tau protein is known to result in increased tau aggregation and destabilization of the microtubules. Once tau aggregation is induced by hyperphosphorylation, they further accumulate as NFTs within neurons, eventually causing neurodegeneration [47, 48].

Dickey et al. reported that Akt and CHIP coregulate tau degradation through coordinated interactions [49]. Akt is a major cellular kinase, which is ubiquitinated and degraded by the tau ubiquitin ligase CHIP, and this largely depends on the Hsp90 complex, while CHIP, a highly conserved ubiquitin ligase that is critical for quality control and stress recovery systems in most cell types, binds with both HSP70 and Hsp90 and thus interacts with and degrades a number of proteins through the HSP70 or Hsp90 scaffold. Akt regulates the CHIP/Hsp90 complex and reduces CHIP-mediated tau ubiquitination and slowed its degradation, while the CHIP/Hsp90 complex can facilitate the degradation of aberrant tau or promote its refolding back into native tau. In addition, they also found that Akt interacted directly with a critical tau kinase, the microtubule affinity-regulating kinase 2 (PAR1/MARK2), whereby Akt could enhance phosphorylation of tau at S262/S356, a site that was not recognized by the CHIP/Hsp90 complex [49].

The major tau kinases are proline-directed protein kinases targeting serine-proline or threonine-proline motifs. Glycogen synthase kinase-3 beta (GSK3*β*) and cyclin-dependent protein kinase-5 (CDK5), proline-directed kinases, are well-known enzymes linked to neurofibrillary pathology in AD, both of which were suggested to cause phosphorylation of tau on pathogenic sites in AD [50, 51]. In addition, the most important regulators of Hsp90 machinery are the cochaperones and the posttranslational modifications of the Hsp90 protein itself, for example, acetylation, nitrosylation, and phosphorylation [52]. For instance, the acetylation of Hsp90 can inhibit the binding of clients to Hsp90 and in that way enhance their client's proteasomal degradation.

**Figure 1:**
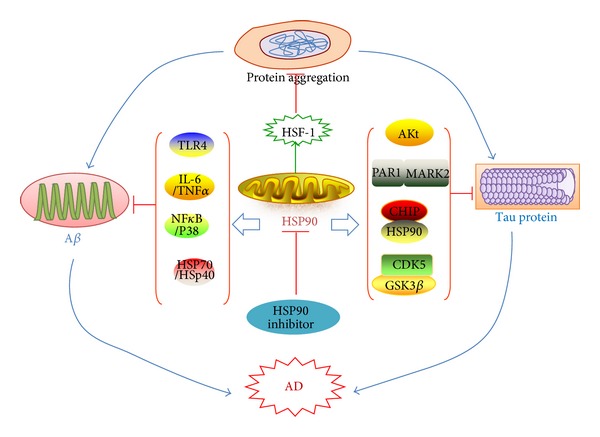
Possible roles of Hsp90 in AD. The two hallmarks of the pathology of AD are the extracellular aggregated A*β* and the intracellular microtubule-associated protein tau. Hsp90 plays a key role in preventing protein misfolding and aggregation. Hsp90 can increase the clearance of A*β* peptides and facilitate A*β* degradation by the activation of TLR4 pathway, the formation of the complex of Hsp90 and HSP70/HSP40, and inducing the production of IL-6 and TNFα. In addition, Akt and CHIP coregulate tau degradation through coordinated interactions. Aberrant activation of kinases such as CDK5 and GSK3*β* can cause phosphorylation of tau. The possible roles of Hsp90 in AD pathogenesis may open a novel therapeutic target for AD.

Tau protein is a client protein for Hsp90 complexes. If the tau protein is in an abnormal or modified form, then it can trigger the recruitment of CHIP protein, to the complex which induces the ubiquitination of tau protein and activates its downstream degradation processes. The upregulation of CHIP expression can attenuate tau aggregation whereas the deletion of CHIP in mice causes the accumulation of tau protein [53, 54] (Figure 1).

## 4. Hsp90 as a Therapeutic Target for AD

So far, Hsp90 has been the most widely tested target for cancer therapy. Hsp90 inhibition is a promising new treatment strategy showing clinical activity in specific tumor types (e.g., ALK-rearranged nonsmall-cell lung cancer and HER2-amplified breast cancer and multiple myeloma). Many oncogenes, including tyrosine kinases, transcription factors, and cell-cycle regulatory proteins, are client proteins of Hsp90. Inhibition of Hsp90 causes client protein degradation via the ubiquitin-proteasome pathway and is a mechanism that might simultaneously downregulate several redundant pathways crucial for cell viability and tumor development, which has resulted in substantial antitumor effects in preclinical models and could potentially prevent the emergence of tumor drug resistance. Inhibition of Hsp90 also binds to the nucleotide-binding site of the N-terminal domain of Hsp90 with higher affinity than ATP; this prevents ATP binding and hydrolysis and eventually leads to enhanced depletion of oncogenic client proteins through ubiquitin-mediated proteasomal degradation [55].

But there are different mechanisms for Hsp90 inhibition as a therapeutic target for the treatment between cancer and AD. Hsp90 inhibition may offer a dual therapeutic approach in AD. On one hand, by reduction of aberrant neuronal protein activity, Hsp90 inhibitor may ameliorate protein hyperphosphorylation and subsequent aggregation. On the other hand, by activation of HSF-1 and the subsequent induction of heat shock proteins, such as Hsp70, Hsp90 inhibitor may redirect neuronal aggregate formation and protect against protein toxicity. Hsp90 can tightly regulate the activity of HSF-1—a master regulator of the heat shock response. In the presence of stress, it dissociates from Hsp90. Once released, HSF-1 is phosphorylated and subsequently trimerizes before entering the nucleus to bind elements that regulate the heat shock response. The HSPs that are induced in response to HSF-1 transcriptional activation include Hsp27, Hsp40, Hsp70, and Hsp90. The expression of these chaperones expands the buffering capacity of the cell and restores protein homeostasis under stressful conditions [56].

Besides, inhibition of Hsp90 in both cellular and mouse models of tauopathies led to reduction of the pathogenic activity of these proteins and resulted in a dose- and time-dependent elimination of aggregated tau [51]. GSK3*β*, CDK5, and Akt are well-known kinases that are responsible for the phosphorylation of tau and are also Hsp90-dependent substrates. p35 and its cleavage product p25 are neuronal proteins that activate CDK5 and are also dependent upon Hsp90 for their activity. Consequently, through Hsp90 inhibition, degradation of these kinases appears to reduce the amount of hyperphosphorylated tau as well as to direct degradation of pathogenic tau species.

Current therapies for AD involve cholinesterase inhibitors and NMDA receptor antagonists, which only slow disease progression [57]. Hsp90 inhibition as a therapeutic target for the treatment of AD has attracted more and more attention. The most classical Hsp90 inhibitor is geldanamycin (GA), a natural product that was developed as an antifungal agent. Radicicol (RDC) was subsequently discovered as an Hsp90 inhibitor shortly after GA, and both GA and RDC have been shown to competitively bind to the N-terminal ATP-binding site. GA and its derivatives most likely exert their activity via two mechanisms: one which occurs through induction of the cytoprotective heat shock response and the other which directs pathogenic proteins to degradation [56]. 17-(Allylamino)-17-demethoxygeldanamycin (17-AAG) was a new derivative of GA, which has the same biological activities but shows less toxicity [8]. 17-AAG reduces the total amount of p-tau and its abnormal aggregation. Dou et al. demonstrated that GA and 17-AAG blocked aberrant tau phosphorylation indirectly by inhibition of the Raf-MEK-ERK pathway, of which Raf is also an Hsp90 client protein, while extracellular signal-regulated kinase (ERK) is known to mediate the activation and stabilization of p-tau [8, 36, 58, 59].

A recent study by Gezen-Ak et al. demonstrated a systemic downregulation of Hsp90 in early and late onset Alzheimer's disease. Serum Hsp90 levels in the EOAD, LOAD, and MCI patients were significantly decreased compared with controls [60]. Moreover, administration of Hsp90 inhibitors could prevent A*β*-induced neurotoxicity by increasing levels of HSP70 and Hsp90 [39, 61]. These results agree with those of a study that investigated the link between Hsp90 and AD [62] and might be suggested as the decreased serum Hsp90 levels is a sign of increasing protein aggregation in AD [60].

These findings suggest that Hsp90 inhibitor might provide a broader, more effective antineurodegenerative therapy than most current drug discovery efforts.

## 5. Conclusions and Future Perspectives

HSPs as molecular chaperones play an important role in the quality control of proteins, and Hsp90 is involved in the folding, activation, and assembly of its client proteins as a ubiquitous molecular chaperone. AD is chronic and progressive, accompanied by the accumulation of intracellular or extracellular protein. Recently, it has been proved that Hsp90 might become a potential therapeutic target for AD. However, the development of Hsp90 for the treatment of AD remains in its infancy; significant investigations are necessary to determine the clinical relevance of Hsp90 modulators for AD treatment. In addition, further research of Hsp90 might provide a detailed approach and novel opportunities to better understand the disease pathogenesis and identify therapeutic targets for these patients. Several years may be spent to achieve the goal in AD treatment, and we need more research to fully understand the biology of Hsp90 in AD. Through a greater understanding of the mechanism between Hsp90 and associated client proteins, there is no doubt that these targets will come to light, and a new generation of AD therapeutics will emerge. Consequently, Hsp90 may represent a unique and exciting therapeutic target for AD in the future.
